# Highly Selective Oxygen/Nitrogen Separation Membrane Engineered Using a Porphyrin-Based Oxygen Carrier

**DOI:** 10.3390/membranes9090115

**Published:** 2019-09-03

**Authors:** Jiuli Han, Lu Bai, Bingbing Yang, Yinge Bai, Shuangjiang Luo, Shaojuan Zeng, Hongshuai Gao, Yi Nie, Xiaoyan Ji, Suojiang Zhang, Xiangping Zhang

**Affiliations:** 1 Beijing Key Laboratory of Ionic Liquids Clean Process, State Key Laboratory of Multiphase Complex Systems, CAS Key Laboratory of Green Process and Engineering, Institute of Process Engineering, Chinese Academy of Sciences, Beijing 100190, China; 2 School of Chemical Engineering, University of Chinese Academy of Sciences, Beijing 100049, China; 3 Zhengzhou Institute of Emerging Industrial Technology, Zhengzhou 450000, China; 4 Energy Engineering, Division of Energy Science, Luleå University of Technology, 97187 Luleå, Sweden

**Keywords:** facilitated transport membranes, cobalt porphyrin, oxygen/nitrogen separation, mixed matrix membrane, selectivity

## Abstract

Air separation is very important from the viewpoint of the economic and environmental advantages. In this work, defect-free facilitated transport membranes based on poly(amide-12-b-ethylene oxide) (Pebax-2533) and tetra(*p*-methoxylphenyl)porphyrin cobalt chloride (T(*p*-OCH_3_)PPCoCl) were fabricated in systematically varied compositions for O_2_/N_2_ separation. T(*p*-OCH_3_)PPCoCl was introduced as carriers that selectively and reversibly interacted with O_2_ and facilitated O_2_ transport in the membrane. The T(*p*-OCH_3_)PPCoCl had good compatibility with the Pebax-2533 via the hydrogen bond interaction and formed a uniform and thin selective layer on the substrate. The O_2_ separation performance of the thin film composite (TFC) membranes was improved by adding a small amount of the T(*p*-OCH_3_)PPCoCl and decreasing the feed pressure. At the pressure of 0.035 MPa, the O_2_ permeability and O_2_/N_2_ selectivity of the 0.6 wt % T(*p*-OCH_3_)PPCoCl/Pebax-2533 was more than 3.5 times that of the Pebax-2533 TFC membrane, which reached the 2008 Robeson upper bound. It provides a candidate membrane material for O_2_/N_2_ efficient separation in moderate conditions.

## 1. Introduction

Air separation is an essential process, in which the obtained oxygen-enriched air can be used to assist combustion by increasing the burning velocities [1], regenerate catalysts in the fluid catalytic cracking [2], and improve indoor air quality [3]; meanwhile, the obtained nitrogen-enriched air can be applied in keeping food fresh, preventing fires, oil recovery, and draining water [4,5]. Currently, cryogenic distillation is the commercial and mature technology for air separation, which can produce a large amount of high purity (>99%) oxygen and nitrogen. However, this process is complex, as well as cost and energy intensive [6]. Pressure swing adsorption (PSA) is another commonly used air separation method that can produce high purity (≈95%) oxygen with medium production, but the larger space, higher investment, and energy consumption of the process are still challenging [7]. Compared with these air separation technologies, the membrane gas separation method is a green and sustainable process because of its continuous production, easy operation, small space, environmental friendliness, and energy conservation [7,8,9,10,11]. Polymeric membranes are usually applied for air separation, but their gas separation performance is always limited by the Robeson upper bound between O_2_ permeability and O_2_/N_2_ selectivity [12]. Therefore, developing membranes with both excellent O_2_ permeability and O_2_/N_2_ selectivity for efficient air separation is highly desired.

Facilitated oxygen transport through membranes is an effective approach to enhance the separation performance or even overcome the Robeson upper bound. These membranes usually contain metal complexes as the oxygen carrier, like cobalt phthalocyanine [13], cobalt Schiff [14,15,16], and cobalt porphyrins [17,18,19,20,21,22,23,24]. They have a reversible interaction with O_2_, facilitating O_2_ transportation prior to other gases in membranes, so that the O_2_ separation performance is improved. Nagar et al. [13] prepared the facilitated transport membrane using cobalt phthalocyanine (CoPc) as the oxygen carrier and studied the effect of CoPc content and feed pressure on the O_2_/N_2_ separation performance. The O_2_ permeance and O_2_/N_2_ selectivity are up to 1.12 GPU (1 GPU = 10^−6^ cm^3^ cm^−2^ s^−1^ cmHg^−1^) and 8.5 at the CoPc content of 1 wt % and the feed pressure of 2 bar. Matsuoka et al. [15] fabricated two kinds of liquid membranes with N,N′-bis(salicylidene)ethylenediamine cobalt-based ionic liquid as the oxygen carrier. At a low feed pressure of 1 kPa, the O_2_/N_2_ separation performance overcomes the 2008 Robeson upper bound. Cobalt porphyrins were also applied as oxygen carriers owing to their high O_2_ affinity, easy preparation, and high stability. Yang et al. [19] investigated the effect of cobalt porphyrins with different substituents and axial ligands on the O_2_ permeability and O_2_/N_2_ ideal selectivity. The results showed the cobalt porphyrin containing an electron-accepting substituent (–Cl) and imidazole as the ligand could increase the O_2_/N_2_ selectivity of the ethyl cellulose membrane. The O_2_ permeability and O_2_/N_2_ selectivity are 12.39 barrer and 4.44, respectively. This is due to the electron-accepting substituent (–Cl) decreasing the electron density at the center cobalt ion via electron-inductive effects and decreasing the oxygen-binding rate, hence increasing the O_2_/N_2_ selectivity [20]. Shinohara et al. [21] prepared cobalt tetraazaporphyrin with a polymeric imidazole ligand and investigated its O_2_ separation performance, similarly showing that the low electron density at the cobalt ion leads to low oxygen-binding affinity and enhances the oxygen-releasing rate constant to elevate the O_2_ separation performance. The O_2_/N_2_ selectivity is up to 28 with a relatively low O_2_ permeability of 2.9 barrer. Shoji et al. [22] developed cobalt porphyrin-Nafion membranes and studied the O_2_/N_2_ separation performance. The results showed that the membrane containing cobalt porphyrin with the electron-accepting group –F (*meso*-tetrakis(pentafluorophenyl)porpyrinatocobalt, CoFPP) has a higher O_2_/N_2_ selectivity than that including *meso*-tetraphenylporphyrinatocobalt (CoTPP) as the carrier because the CoFPP has a lower oxygen-binding affinity than the CoTPP. Choi et al. [24] investigated the O_2_/N_2_ separation performance of the poly(n-butyl methacrylate)-cobalt tetraphenylporphyrin composite hollow fiber membrane and the O_2_ permeability and O_2_/N_2_ selectivity are ≈5.2 barrer and 3.2, respectively. However, both the metalloporphyrin and polymer are solids. Their surface compatibility is still challenged and improved. Obviously, good compatibility is beneficial to the dispersion of the metalloporphyrin, increasing the O_2_ transport efficiency and then improving the membrane separation performance.

In this work, we developed a novel thin-film composite (TFC) membrane for O_2_/N_2_ separation, which was fabricated using the cobalt porphyrin (T(*p*-OCH_3_)PPCoCl) as the oxygen carrier, Pebax-2533 as the polymer matrix, and macroporous PVDF as the support. The substitute –OCH_3_ connected to porphyrin was favorable for the compatibility between cobalt porphyrin and Pebax-2533. The cobalt porphyrin with –Cl as the axial ligand could decrease the electron density at the cobalt ion, and decrease the oxygen-binding affinity, hence enhance the oxygen-releasing rate to achieve the effective O_2_/N_2_ separation. The prepared TFC membranes were characterized, and the existing interaction between T(*p*-OCH_3_)PPCoCl and O_2_ was proved via simulation. The influence of T(*p*-OCH_3_)PPCoCl with different contents and different pressures on the O_2_ permeability, N_2_ permeability, and O_2_/N_2_ selectivity was systematically studied.

## 2. Materials and Methods

### 2.1. Materials

**Chart 1:**
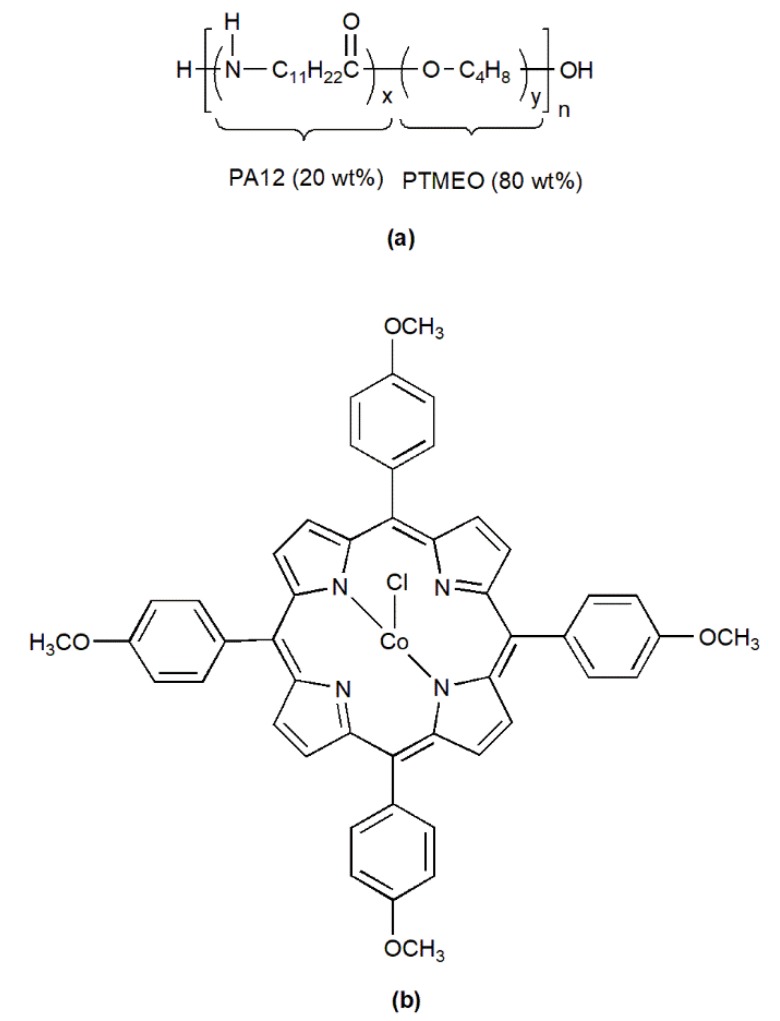
Chemical structures of Pebax-2533 (**a**) and T(*p*-OCH_3_)PPCoCl (**b**).

The PVDF support membrane with a pore size of 0.1 μm and an average thickness of 100 μm was purchased from Hangzhou Anow Microfiltration Co., Ltd. (Hangzhou, China). Pebax-2533 containing 80 wt % of poly(tetramethylene oxide) (PTMEO) and 20 wt % of nylon 12 (PA12) was provided by Arkema (Paris, France), and its structure is shown in Chart 1a. The N_2_ (99.999%) and O_2_ (99.999%) were supplied by Beijing Beiwen Gas Factory (Beijing, China). Hydrochloric acid, anhydrous methanol, and ethanol were purchased from Beijing Chemical Works (Beijing, China). *o*-nitrotoluene, *p*-anisaldehyde, propionic acid, and acetic acid were supplied by Aladdin (Shanghai, China). Pyrrole and anhydrous cobalt dichloride (CoCl_2_) was purchased from Sinopharm Chemical Reagent Co., Ltd. (Shanghai, China). Dimethylformamide (DMF) was provided by Xilong Scientific Co., Ltd. (Guangdong, China). Deionized water was used throughout the study.

### 2.2. Synthesis of Cobalt Porphyrin

Tetra(*p*-methoxylphenyl)porphyrin (T(*p*-OCH_3_)PP) was prepared according to the literature [25,26] with some revisions. In a three-necked flask, propionic acid (20 mL), acetic acid (10 mL), and *o*-nitrotoluene (10 mL) were added. The mixture was heated to reflux, and then *p*-anisaldehyde (10 mmol) was added into the refluxing mixture and stirred for 5–6 min. Pyrrole (10 mmol) dissolved in *o*-nitrotoluene (10 mL) was added dropwise and reacted for 1 h. The mixture was allowed to cool to 60 °C, and then 30 mL of methanol was added slowly and set aside for recrystallization. The blue crystals were filtered and washed three times with water and methanol. The resulting T(*p*-OCH_3_)PP product (0.34 g, 18.5% yield) was obtained and dried at 60 °C under vacuum for 8 h. ^1^H NMR (600 MHz, CDCl_3_) δ (ppm): −2.75 (s, 2H); 4.10 (s, 12H); 7.28 (d, *J* = 8.4 Hz, 8H); 8.12 (d, *J* = 8.4 Hz, 8H); 8.86 (s, 8H).

Tetra(*p*-methoxylphenyl)porphyrin cobalt chloride (T(*p*-OCH_3_)PPCoCl) (Chart 1b) was synthesized according to the literature [27]. T(*p*-OCH_3_)PP (147 mg, 0.2 mmol), DMF(30 mL) and acetic acid (3 mL) were added into a three-necked flask and heated to reflux for 30 min. Then, CoCl_2_ (156 mg, 1.2 mmol) was added with five portions and reacted for 5 h. After the mixture was cooled down to room temperature, hydrochloric acid aqueous solution (40 mL, ≈19 wt %) was poured into the flask and held overnight for recrystallization. The crystals were filtered, washed thoroughly with hydrochloric acid aqueous solution (200 mL, ≈8 wt %), and dried at 60 °C under vacuum for 8 h to obtain purple crystals of T(*p*-OCH_3_)PPCoCl (160 mg, yield 97%).

### 2.3. Membrane Preparation

Free-standing Pebax-2533 membrane and T(*p*-OCH_3_)PPCoCl/Pebax-2533 mixed matrix membranes (MMMs) were prepared using a solution-casting method. Pebax-2533 pellets were dissolved in ethanol (10 wt %) with magnetic stirring at 80 °C for 3 h. A predetermined amount of T(*p*-OCH_3_)PPCoCl was then added and stirred at 40 °C for 2 h. After that, the solution was cast onto a glass dish and dried at room temperature for 24 h to form a sheet of membrane, which was further dried under vacuum at 60 °C for 8 h.

TFC membranes of T(*p*-OCH_3_)PPCoCl/Pebax-2533 on PVDF supports were prepared using a dip-coating method. Pre-weighed amounts of Pebax-2533 pellets were dissolved in hot ethanol at 80 °C to obtain a homogeneous solution (2 wt %). Then, T(*p*-OCH_3_)PPCoCl was added and the mixture was stirred continuously at 40 °C until it became homogeneous. The solution was degassed and poured into a glass cell. After that, the prefixed substrate PVDF on a glass pane was vertically immersed into the glass cell with the T(*p*-OCH_3_)PPCoCl/Pebax-2533 solution and then dried at ambient temperature for 2 h. Finally, the composite membranes were dried in a vacuum oven at 60 °C for 8 h to remove any solvent residue.

### 2.4. Characterization Methods

Ultraviolet-visible (UV-Vis) spectra of T(*p*-OCH_3_)PP and T(*p*-OCH_3_)PPCoCl were recorded in toluene on a Shimadzu UV-2550 spectrophotometer (Shimadzu Corporation, Kyoto, Japan) in the range of 350–680 nm. The Fourier transform infrared (FTIR) spectrum of T(*p*-OCH_3_)PPCoCl and the attenuated total reflectance Fourier transform infrared (ATR-FTIR) spectra of Pebax-2533 and MMMs were obtained using a Thermo Nicolet 380 spectrometer (Thermo Electron Corporation, Madison, WI, USA) in the range of 650–4000 cm^−1^ under ambient conditions. Wide angle X-ray diffraction (WAXRD) patterns of the MMMs were measured using a Smartlab (9 kW) diffractometer (Rigaku Corporation, Tokyo, Japan) at a scan rate of 15° min^−1^; the *d*-spacing values were calculated based on the diffraction peak maxima using Bragg’s equation. The surface and cross-sectional morphologies of the TFC membranes were observed using SU8020 scanning electron microscopy (SEM) (Hitachi High-Technologies Corporation, Tokyo, Japan). Before scanning, the membranes were fractured in liquid nitrogen and sputtered with gold.

The O_2_ and N_2_ permeation tests were carried out at ambient temperature using the standard constant-pressure, variable-volume method [28]. The feed pressure varied from 0.035 to 0.8 MPa in gauge mode, and the permeate pressure was kept at atmospheric pressure. Gas permeability was calculated using the following equation:

(1) $$P=\frac{Ql}{tA(p_{1}-p_{2})}$$

where P is the gas permeability (barrer, 1 barrer = 10^−10^ (cm^3^ (STP) cm cm^−2^ s^−1^ cmHg^−1^), Q is the volume of permeated gas (cm^3^ (STP)), l is the film thickness (cm), t is the permeation time (s), A is the effective membrane area (cm^2^), and $p_{1}$ and $p_{2}$ are the upstream and permeate side pressures (cmHg), respectively. The ideal selectivity (S) is defined as the ratio of the pure gas permeability of the faster gas O_2_ ($P_{A}$) over that of the slower permeant N_2_ ($P_{B}$):

(2) $$S=\frac{P_{A}}{P_{B}}$$

### 2.5. Molecular Modeling

Molecular geometries (O_2_, N_2_, and T(*p*-OCH_3_)PPCoCl) were optimized using density functional theory (DFT) calculations with Gaussian 09 software (Gaussian, Inc., Wallingford, CT, USA) [29]. The formation of complexes between T(*p*-OCH_3_)PPCoCl and N_2_/O_2_ with several different structures were fabricated by locating N_2_ or O_2_ at different positions around T(*p*-OCH_3_)PPCoCl, and the energy minimizations of all complexes were implemented to determine the optimized geometry. All of the optimizations were explored at the B3LYP/6-311G (*d*, *p*) level. The interaction energies between T(*p*-OCH_3_)PPCoCl and N_2_/O_2_ were calculated at the same level.

## 3. Results and Discussion

### 3.1. Synthesis and Characterization of T(p-OCH_3_)PPCoCl

**Figure 1:**
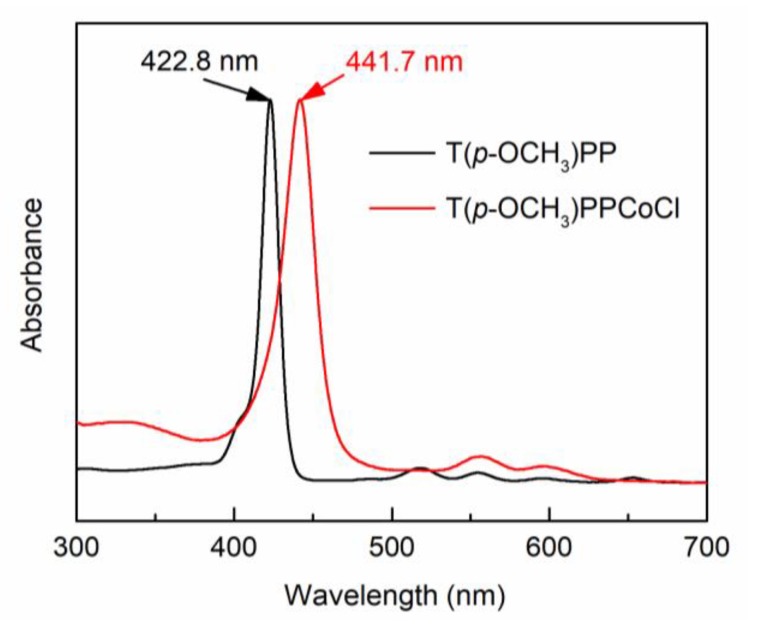
UV-vis spectra of T(*p*-OCH_3_)PP and T(*p*-OCH_3_)PPCoCl.

T(*p*-OCH_3_)PP was prepared in a mixed solvent of propionic acid, acetic acid, and *o*-nitrotoluene under an air atmosphere. Propionic acid and acetic acid were used as catalysts for the condensation of pyrrole and *p*-anisaldehyde to obtain porphyrinogen, which was further oxidized by the oxygen (air) and *o*-nitrotoluene to produce T(*p*-OCH_3_)PP with a yield of 18.5%. Compared with other metalloporphyrins, cobalt porphyrin has a higher stability [30]. Therefore, T(*p*-OCH_3_)PPCoCl was used as the O_2_ carrier and prepared in DMF with a high yield (97%). The obtained T(*p*-OCH_3_)PP and T(*p*-OCH_3_)PPCoCl were characterized using UV-vis and the spectra are shown in Figure 1. T(*p*-OCH_3_)PP exhibited a characteristic major Soret band at 422.8 nm owing to the π→π* transition, and four visible Q bands at 518.2, 554.7, 595.2, and 653.3 nm. The Soret band of T(*p*-OCH_3_)PPCoCl shifted to a longer wavelength of 441.7 nm and two Q bands emerged at 556.8 nm and 596.1 nm. The decreased amount of Q bands can be ascribed the metalation of T(*p*-OCH_3_)PP, which increased the molecular symmetry [31].

### 3.2. Membranes Properties and Characterizations

**Figure 2:**
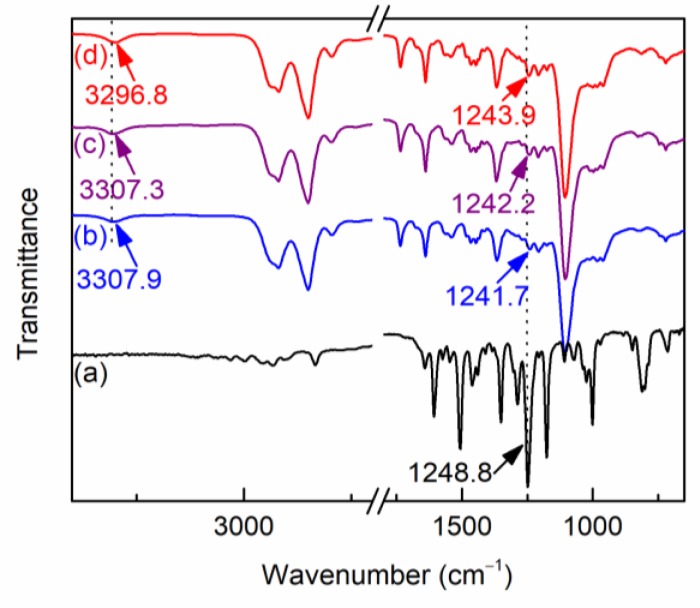
FTIR spectra of T(*p*-OCH_3_)PPCoCl (**a**), Pebax-2533 membrane (**b**), 0.6 wt % T(*p*-OCH_3_)PPCoCl/Pebax-2533 membrane (**c**) and 2 wt % T(*p*-OCH_3_)PPCoCl/Pebax-2533 membrane (**d**).

The interaction between Pebax-2533 matrix and T(*p*-OCH_3_)PPCoCl was investigated using FTIR, and the spectra of T(*p*-OCH_3_)PPCoCl, neat Pebax-2533 membrane, and T(*p*-OCH_3_)PPCoCl/Pebax-2533 MMMs are given in Figure 2. The absorption peaks at 2834.0, 1606.2, 1351.2, 1248.8, and 1001.7 cm^−1^ for T(*p*-OCH_3_)PPCoCl were assigned to C–H, C=C, C=N, –C–O–C, and Co–N stretching vibrations, respectively. The spectrum of neat Pebax-2533 membrane was in accordance with the literature [32,33], in which the bands at 3307.9, 1734.7, 1638.2, and 1104.0 cm^−1^ represent the N–H, C=O, H–N–C=O, and C–O stretching, respectively. The peak at 1248.8 cm^−1^ corresponds to the –C–O–C stretching vibration, which showed an increasing intensity with the increasing of the T(*p*-OCH_3_)PPCoCl content in the MMMs, as expected. More importantly, the –C–O–C stretching vibration in T(*p*-OCH_3_)PPCoCl shifted to a longer wavelength from 1248.8 to 1243.9 cm^−1^ in MMMs and the N–H stretching vibration in Pebax-2533 similarly shifted to a longer wavelength from 3307.9 to 3296.8 cm^−1^, confirming substantial hydrogen bonding was formed in the composite membranes [34]. The formation of hydrogen bonding could improve the affinity between T(*p*-OCH_3_)PPCoCl and Pebax-2533, thus avoiding interfacial defects.

**Figure 3:**
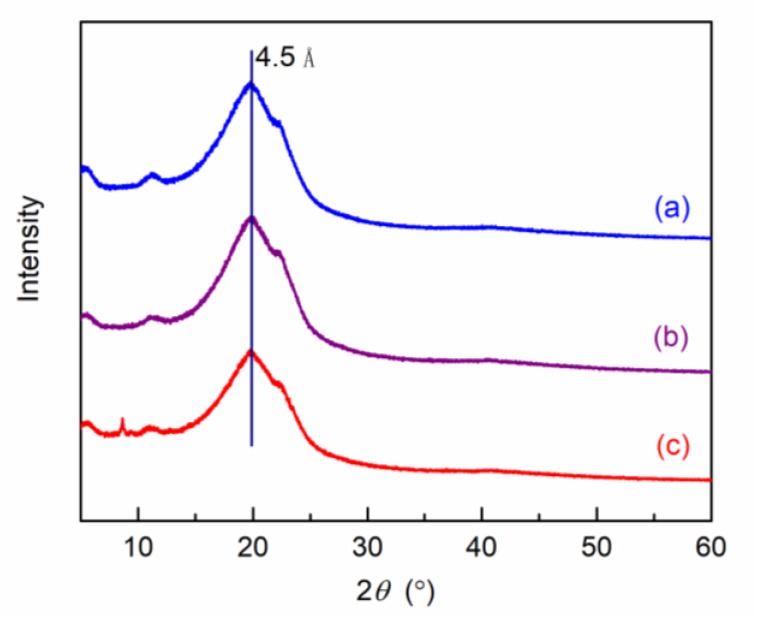
XRD spectra of Pebax-2533 membrane (**a**), 0.6 wt % T(*p*-OCH_3_)PPCoCl/Pebax-2533 membrane (**b**), and 2 wt % T(*p*-OCH_3_)PPCoCl/Pebax-2533 membrane (**c**).

To investigate the effect of T(*p*-OCH_3_)PPCoCl on the chain packing structure of Pebax-2533, XRD patterns of the Pebax-2533 membrane, 0.6 wt % T(*p*-OCH_3_)PPCoCl/Pebax-2533 MMM and 2 wt % T(*p*-OCH_3_)PPCoCl/Pebax-2533 MMM were measured, as shown in Figure 3. The neat Pebax-2533 membrane had a broad peak located at 2*θ* = 19.8°, which corresponds to a *d*-spacing value of 4.5 Å. The broad halo was identified as the inter-chain distance of amorphous regions [35,36,37]. Adding T(*p*-OCH_3_)PPCoCl into Pebax-2533 matrix did not change the polymer chain packing structure, as evidenced in Figure 3, where the *d*-spacing values of the MMMs were the same as that of the pure Pebax-2533 membrane. No crystal peak of T(*p*-OCH_3_)PPCoCl was observed in the WAXD patterns. This could have originated from a small weight fraction of the T(*p*-OCH_3_)PPCoCl and suggests a good compatibility in the T(*p*-OCH_3_)PPCoCl/Pebax-2533 composite membranes.

**Figure 4:**
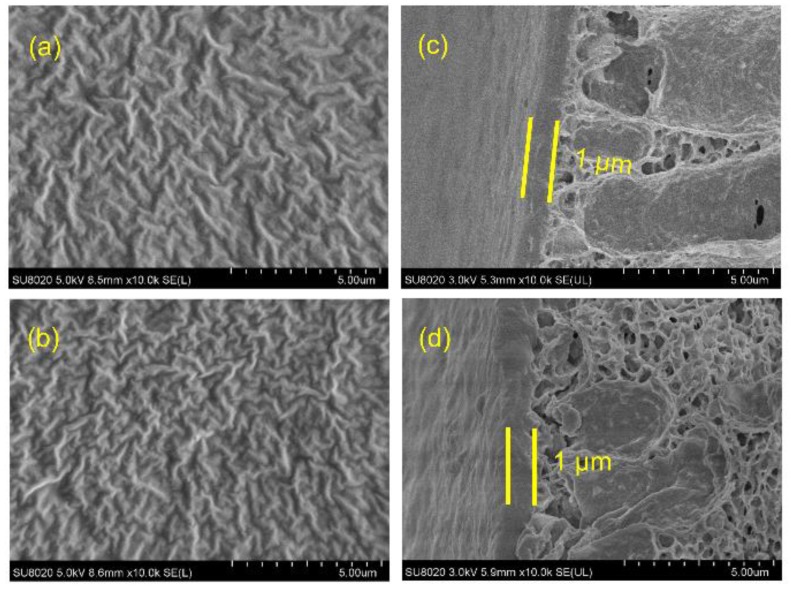
The surface SEM images of (**a**) Pebax-2533 membrane and (**b**) 0.6 wt % T(*p*-OCH_3_)PPCoCl/Pebax-2533 membrane, and the cross-section SEM images of (**c**) Pebax-2533 membrane and (**d**) 0.6 wt % T(*p*-OCH_3_)PPCoCl/Pebax-2533 membrane.

Figure 4 exhibits the surface and cross-sectional morphologies of the Pebax-2533 membrane and 0.6 wt % T(*p*-OCH_3_)PPCoCl/Pebax-2533 TFC membrane. SEM images display homogeneous surfaces, and the cross-sectional view (Figure 4c) of Pebax-2533 shows two layers, i.e., the top selective layer with a film thickness of 1 μm and the PVDF substrate. As shown in Figure 4b,d, no particulate clusters were observed, indicating that T(*p*-OCH_3_)PPCoCl was well dispersed at the molecular level in the Pebax-2533 matrix without obvious agglomerations.

### 3.3. Molecular Modeling of O_2_/N_2_ and T(p-OCH_3_)PPCoCl Interactions

**Figure 5:**
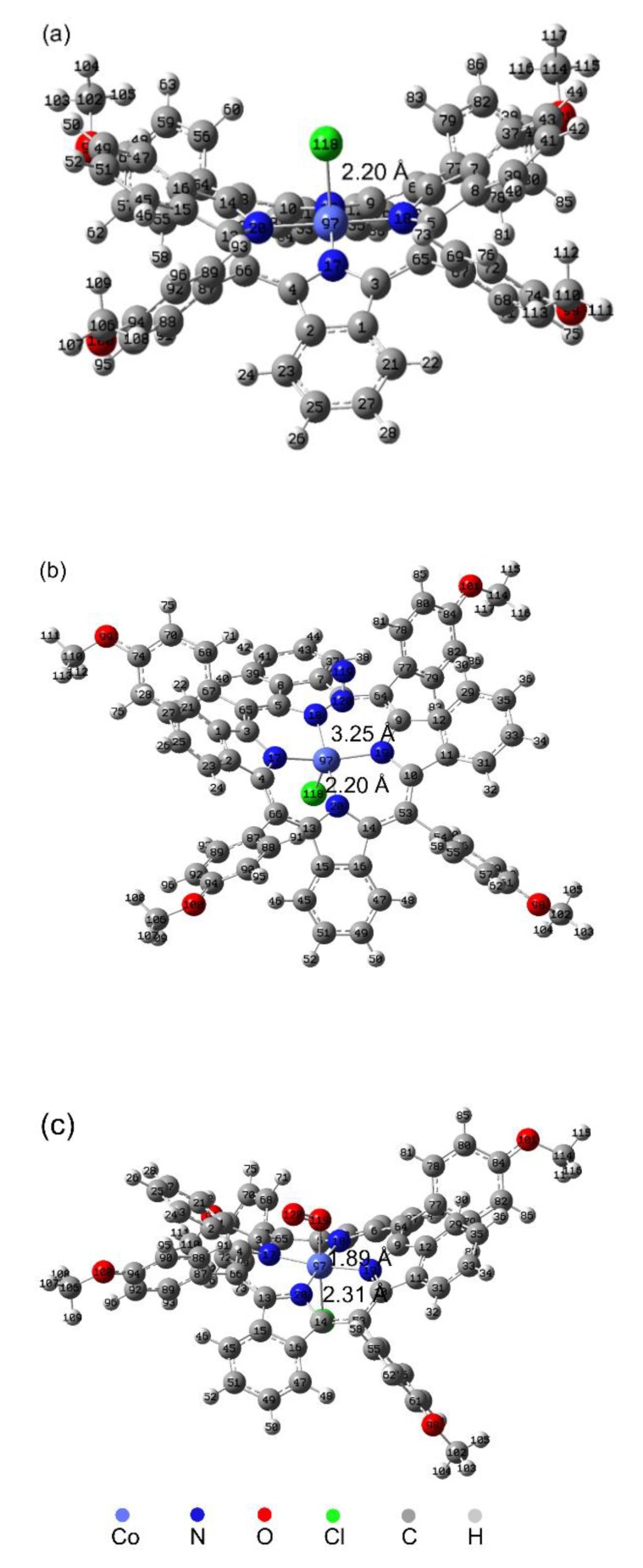
Optimized structures of (**a**) T(*p*-OCH_3_)PPCoCl, (**b**) T(*p*-OCH_3_)PPCoCl–N_2_ complex, and (**c**) T(*p*-OCH_3_)PPCoCl–O_2_ complex at the B3LYP/6-311G (*d*, *p*) level.

The optimized structures of the T(*p*-OCH_3_)PPCoCl and the interaction between T(*p*-OCH_3_)PPCoCl and gas molecules were studied using molecular modeling, and the results are shown in Figure 5. T(*p*-OCH_3_)PPCoCl exhibited a three-dimensional and distorted structure (Figure 5a), and it may have increased the fractional free volume and gas permeability of the membranes by disrupting the polymer chain packing. In the T(*p*-OCH_3_)PPCoCl–N_2_ complex (Figure 5b), the distance between 97Co and 120N was 3.25 Å, and there was no obvious bond, confirming there was no interaction between T(*p*-OCH_3_)PPCoCl and N_2_. However, in the T(*p*-OCH_3_)PPCoCl–O_2_ complex (Figure 5c), O_2_ interacted with the central cobalt ion of T(*p*-OCH_3_)PPCoCl to form a six-coordinate complex (Figure 5c). The distance of 97Co–119O was 1.89 Å, which is the same as the length of the reported Co–O coordinate bond [38], showing the coordinate bond was formed between 97Co and 119O. It indicates there was an interaction between T(*p*-OCH_3_)PPCoCl and O_2_. At the same time, the distance of 97Co–118Cl (2.31 Å) in the T(*p*-OCH_3_)PPCoCl–O_2_ complex was longer than that (2.20 Å) in T(*p*-OCH_3_)PPCoCl and shows the strength of the 97Co–118Cl bond decreased during the T(*p*-OCH_3_)PPCoCl–O_2_ complex formation. Their interaction energies between T(*p*-OCH_3_)PPCoCl and N_2_ or O_2_ estimated using quantum chemical calculations were −1.03 kcal/mol and −23.54 kcal/mol, respectively. It shows the interaction between T(*p*-OCH_3_)PPCoCl and O_2_ was higher than that between T(*p*-OCH_3_)PPCoCl and N_2_ and they may be reversible chemical and physical interactions separately. The reversible interaction makes T(*p*-OCH_3_)PPCoCl selectively adsorb oxygen and facilitate its transport in the membrane.

### 3.4. Gas Permeation Properties

**Figure 6:**
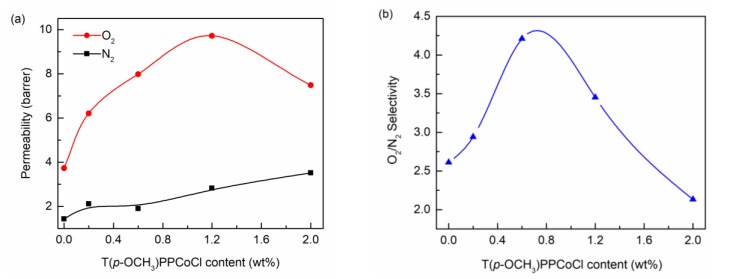
Effect of T(*p*-OCH_3_)PPCoCl content on gas permeability (**a**) and O_2_/N_2_ selectivity (**b**).

The influence of the T(*p*-OCH_3_)PPCoCl content on O_2_ and N_2_ permeability and O_2_/N_2_ selectivity were systematically investigated. It can be seen from Figure 6a that the N_2_ permeability increased with the increasing T(*p*-OCH_3_)PPCoCl content. Given that there was almost no interaction between N_2_ and T(*p*-OCH_3_)PPCoCl (interaction energy of −1.03 kcal/mol), the increase of the N_2_ permeability was mainly due to the increase of the free volume resulting from adding the twisted T(*p*-OCH_3_)PPCoCl into the membrane. However, the O_2_ permeability first increased and then decreased with the increasing T(*p*-OCH_3_)PPCoCl content. The O_2_ permeability of the T(*p*-OCH_3_)PPCoCl/Pebax-2533 TFC membrane reached a maximum value of 9.7 barrer at 1.2 wt % T(*p*-OCH_3_)PPCoCl content, which is about 2.6 times that of a pure Pebax-2533 membrane. The suitable interaction energy of −23.54 kcal/mol between O_2_ and T(*p*-OCH_3_)PPCoCl made T(*p*-OCH_3_)PPCoCl selectively adsorb O_2_ and then release O_2_ quickly, thus facilitating the O_2_ transport in the membrane and enhancing the O_2_ separation performance. When the T(*p*-OCH_3_)PPCoCl content was between 0–1.2 wt %, there were more T(*p*-OCH_3_)PPCoCl molecules to interact with O_2_ and facilitate its transport in the membrane with the increasing of T(*p*-OCH_3_)PPCoCl content. Meanwhile, the addition of T(*p*-OCH_3_)PPCoCl increased the free volume of the membranes. These led to an increase of the O_2_ permeability. However, when further increasing the T(*p*-OCH_3_)PPCoCl content, the O_2_ permeability decreased, probably owing to the aggregation of T(*p*-OCH_3_)PPCoCl particles. The O_2_/N_2_ selectivity also increased initially and then decreased with the T(*p*-OCH_3_)PPCoCl content increase (Figure 6b). The T(*p*-OCH_3_)PPCoCl/Pebax-2533 TFC membrane with the T(*p*-OCH_3_)PPCoCl content of 0.6 wt % exhibited the maximum O_2_/N_2_ selectivity of 4.2 and a relatively high O_2_ permeability of 8.0 barrer.

**Figure 7:**
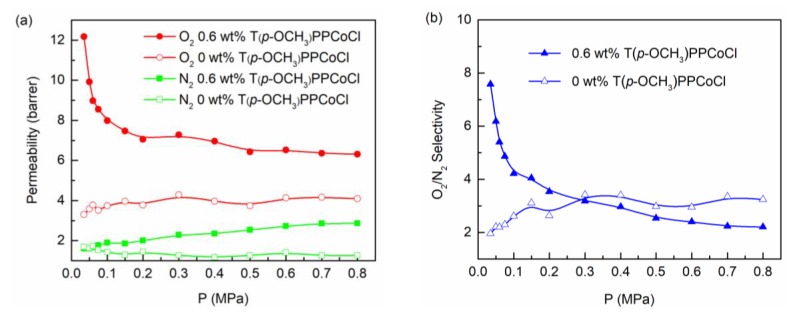
Effect of the feed pressure on gas permeability (**a**) and O_2_/N_2_ selectivity (**b**).

Gas feed pressure also has a great influence on the O_2_ separation performance, and the results of the investigation is shown in Figure 7a,b. The O_2_ and N_2_ permeability of the membrane without T(*p*-OCH_3_)PPCoCl almost remained constant when varying the feed pressure, indicating the change of pressure had no obvious influence. For the TFC membrane containing 0.6 wt % T(*p*-OCH_3_)PPCoCl, the N_2_ permeability increased slightly with the increasing feed pressure. The O_2_ permeability also remained constant when the feed pressure was higher than 0.1 MPa, but interestingly, the O_2_ permeability increased significantly from 8.0 barrer to 12.2 barrer, and the O_2_/N_2_ selectivity increased from 4.2 to 7.6 by lowering the feed pressure from 0.1 MPa to 0.035 MPa. This indicates that the lower pressure was beneficial for O_2_ separation because the chemical interaction between O_2_ and the carrier was predominant at low feed pressure [39], hence promoting the oxygen transport by T(*p*-OCH_3_)PPCoCl.

**Figure 8:**
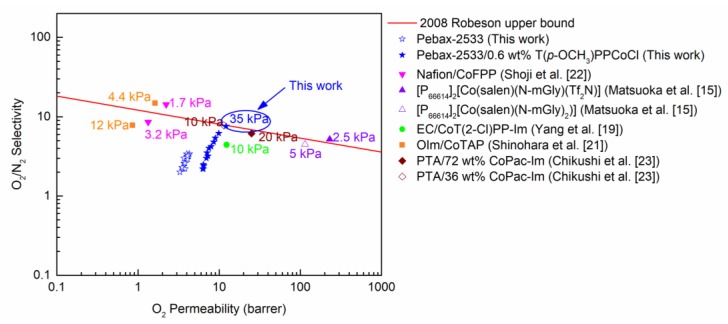
Performance comparison with the Robeson upper bound.

The O_2_/N_2_ separation performances of the T(*p*-OCH_3_)PPCoCl/Pebax-2533 TFC membrane were plotted against the 2008 Robeson upper bound, as shown in Figure 8. Compared with the membrane without T(*p*-OCH_3_)PPCoCl, the adding of T(*p*-OCH_3_)PPCoCl could enhance the separation performance mainly because T(*p*-OCH_3_)PPCoCl could efficiently facilitate O_2_ transport in the membrane, as well as the non-planar T(*p*-OCH_3_)PPCoCl increasing the gas permeability by increasing the fractional free volume. As the feed pressure was reduced, the separation performance was promoted gradually. The O_2_ permeability and O_2_/N_2_ selectivity were 12.2 barrer and 7.6, respectively, at 0.035 MPa, which is near the 2008 Robeson upper bound. For the facilitated membranes, lowering the feed pressure could enhance the separation performance. However, the separation performance of the 0.6 wt % T(*p*-OCH_3_)PPCoCl/Pebax-2533 TFC membrane could reach the 2008 upper bound at a feed pressure of 35 kPa, which is higher than the feed pressures (<20 kPa) of the reported lectures [15,19,21,22,23]. It shows the separation performance of the membrane could more easily reach the upper bound than the reported membranes by reducing the feed pressure. The separation performances of the reported membranes are presented in Table 1 for comparison. The data shows the 0.6 wt % T(*p*-OCH_3_)PPCoCl/Pebax-2533 TFC membrane had satisfactory separation performance. Its O_2_/N_2_ selectivity was lower than only that of the 1 wt % CoPc/Pebax-1657 membrane and 20 wt % CoFPP Nafion membrane, but its O_2_ permeance was much higher than theirs. Thus, the T(*p*-OCH_3_)PPCoCl/Pebax-2533 TFC membrane is a promising candidate for O_2_/N_2_ separation.

## 4. Conclusions

T(*p*-OCH_3_)PPCoCl/Pebax-2533 TFC membranes with a thin and defect-free active layer were prepared. The T(*p*-OCH_3_)PPCoCl and Pebax-2533 had good compatibility due to the formation of the hydrogen bond, improving the dispersion of the T(*p*-OCH_3_)PPCoCl in Pebax-2533. T(*p*-OCH_3_)PPCoCl as an oxygen carrier could not only facilitate oxygen transport due to the reversible interaction between T(*p*-OCH_3_)PPCoCl and O_2_, but also increase the membrane free volume, enhancing the O_2_ and N_2_ permeability and O_2_/N_2_ selectivity. The T(*p*-OCH_3_)PPCoCl content and feed pressure had a great influence on membrane separation performance. The 0.6 wt % T(*p*-OCH_3_)PPCoCl/Pebax-2533 TFC membrane exhibited a better O_2_ permeability of 8.0 barrer and a O_2_/N_2_ selectivity of 4.2 than that of the TFC membrane without T(*p*-OCH_3_)PPCoCl (3.7 barrer and 2.6) at 0.1 MPa. Decreasing the feed pressure was beneficial for the O_2_ separation in the membrane. The O_2_ permeability and O_2_/N_2_ selectivity significantly increased to 12.2 barrer and 7.6 at 0.035 MPa, which is up to the 2008 Robeson upper bound.

## Figures and Tables

**Table 1:** Comparison of the separation performance of the reported membranes with this work.

Membrane	P (bar)	T (°C)	S_O_2_/N_2__	P_O_2__ (barrer) ^a^	Ref.
1–15 wt % MgPc/PEI	12.8	25	≈2.3–5.4	≈0.42–0.13 GPU ^b^	[40]
1–15 wt % CoPc/PEI	12.8	25	≈4.8–1.9	≈0.25–1.13 GPU	[40]
1 wt % cosalen/PU	1	5–35	≈4.2–3.0	≈4.2–13.2	[41]
20 wt % CoFPP/Nafion	1–2	25	≈14.2–2.0	≈2.2–0.3	[22]
SiO_2_–PVP–salcomine	-	25–150	1.4–6.1	0.35–1.48 GPU	[14]
1 wt % CoPc/Pebax-1657	2–8	25	≈8.5–5.5	≈1.12–0.93 GPU	[13]
0.6 wt % T(*p*-OCH_3_)PPCoCl/Pebax-2533	0.35–8	18	7.6–2.2	12.2–6.3	This study

^a^ 1 barrer = 10^−10^ cm^3^ cm cm^−2^ s^−1^ cmHg^−1^; ^b^ 1 GPU = 10^−6^ cm^3^ cm^−2^ s^−1^ cmHg^−1^ = 3.38 × 10^−10^ mol m^−2^ s^−1^ Pa^−1^.

## Abbreviations

Pebax	poly(amide-12-b-ethylene oxide)
T(*p*-OCH_3_)PPCoCl	tetra(*p*-methoxylphenyl)porphyrin cobalt chloride
PVDF	polyvinylidene fluoride
TFC	thin film composite
CoPc	cobalt phthalocyanine
CoFPP	*meso*-tetrakis(pentafluorophenyl)porpyrinatocobalt
CoTPP	*meso*-tetraphenylporphyrinatocobalt
T(*p*-OCH_3_)PP	tetra(*p*-methoxylphenyl)porphyrin
MMMs	mixed matrix membranes
MgPc	magnesium phathalocyanine
PEI	polyetherimide
PU	polyurethane
SiO_2_–PVP–salcomine	SiO_2_–poly(N-vinylpyrrolidone)–(N,N-disalicylideneethylenediaminato)cobalt
[P_66614_]_2_[Co(salen)(N-mGly)(Tf_2_N)]	[trihexyl(tetradecyl)phosphonium]_2_[N,N-bis(salicylidene)ethylenediamine cobalt(N-methylglycinate)(bis(trifluoromethanesulfonyl)imid)]
[P_66614_]_2_[Co(salen)(N-mGly)_2_]	[trihexyl(tetradecyl)phosphonium]_2_[N,N-bis(salicylidene)ethylenediamine cobalt(N-methylglycinate)_2_]
EC	ethyl cellulose
CoT(2-Cl)PP	cobalt(II) *meso*-tetrakis(2-chlorophenyl) porphyrin
Im	imidazole
OIm	poly[(octyl methacrylate)-co-vinylimidazole]
CoTAP	cobalt tetra-*tert*-butyltetraazaporphyrin
CoPac	5-(4-benzylacetoacetate)-10, 15, 20-triphenylcobaltporphyrin
PTA	pentaerythritol tetraacrylate
